# Survival After Minimally Invasive vs Open Surgery for Pancreatic Adenocarcinoma

**DOI:** 10.1001/jamanetworkopen.2022.48147

**Published:** 2022-12-22

**Authors:** Halit Topal, Raymond Aerts, Annouschka Laenen, André Collignon, Joris Jaekers, Joachim Geers, Baki Topal

**Affiliations:** 1 Department of Visceral Surgery, University Hospitals KU Leuven, Leuven, Belgium; 2 Department of Biostatistics and Statistical Bioinformatics Center, University Hospitals KU Leuven, Leuven, Belgium; 3 Department of Management Information & Reporting, University Hospitals KU Leuven, Leuven, Belgium

## Abstract

**Importance:**

Only a few high-volume centers have reported on long-term oncologic outcomes after minimally invasive pancreatic surgery (MIPS) for pancreatic adenocarcinoma, but none of them have shown superior long-term overall survival (OS) compared with open pancreatic surgery (OPS).

**Objective:**

To study long-term survival after MIPS and OPS with curative intent among patients with pancreatic adenocarcinoma.

**Design, Setting, and Participants:**

This comparative effectiveness study used a retrospective analysis of a prospectively maintained electronic database of patient data collected between January 2010 and December 2019. Consecutive patients from a high-volume pancreatic cancer referral center were included. Data analysis was conducted from March to October 2022. Median follow-up time was 56.8 months.

**Exposures:**

Patients were matched using propensity score models to study long-term survival.

**Main Outcomes and Measures:**

Survival outcomes were analyzed using the Cox proportional hazards model. Variables used for propensity score correction were TNM stage, tumor dimension, lymph node status, type of operation, simultaneous vascular resection, neoadjuvant chemotherapy, adjuvant chemotherapy, sex, age, and American Society of Anesthesiologists score. Additional corrections were made for year of surgery and type of adjuvant chemotherapy.

**Results:**

After propensity score matching the sample of 396 patients, there were 198 patients in the MIPS group (89 [44.9%] men; median [range] age, 68 [32-87] years) and 198 in the OPS group (94 [47.5%] men; median [range] age, 67 [39-84] years). Median OS in the MIPS group was 30.7 (95% CI, 26.2-36.8) months compared with 20.3 (95% CI, 17.6-23.5) months after OPS (hazard ratio [HR], 0.70; 95% CI, 0.56-0.87; *P* = .002). Median disease-free survival (DFS) after MIPS vs OPS was 14.8 (95% CI, 11.8-17.0) months vs 10.7 (95% CI, 9.0-12.1) months (HR, 0.71; 95% CI, 0.57-0.89; *P* = .003). Additional corrections for year of surgery and type of adjuvant chemotherapy showed better OS (year of surgery: HR, 0.74; 95% CI, 0.57-0.96; *P* = .02; adjuvant chemotherapy: HR, 0.71; 95% CI, 0.56-0.90; *P* = .005) and DFS (year of surgery: HR, 0.77; 95% CI, 0.59-0.99; *P* = .04; adjuvant chemotherapy: HR, 0.72; 95% CI, 0.57-0.92; *P* = .009) for patients undergoing minimally invasive vs open surgery.

**Conclusions and Relevance:**

In this study of 396 patients with borderline resectable and resectable pancreatic adenocarcinoma, MIPS was associated with better OS and DFS than OPS. Centralization of MIPS should be stimulated, and pancreatic surgeons should be encouraged to pass the learning curve before implementing MIPS for pancreatic adenocarcinoma in daily clinical practice.

## Key Points

**Question:**

Is minimally invasive pancreatic surgery (MIPS) associated with better long-term survival compared with open pancreatic surgery (OPS) among patients with pancreatic adenocarcinoma?

**Findings:**

In this study of 396 patients, the median overall and disease-free survival after MIPS was 30.7 months and 14.8 months, respectively, vs 20.3 months and 10.7 months, respectively, after OPS.

**Meaning:**

These findings suggest that MIPS for pancreatic adenocarcinoma, compared with OPS, may offer better overall and disease-free survival.

## Teaser

This comparative effectiveness study evaluates long-term survival after minimally invasive and standard open surgery with curative intent among patients with pancreatic adenocarcinoma.

## Introduction

Pancreatic ductal adenocarcinoma or pancreatic cancer (PC) is one of the most lethal cancers, with an overall 5-year survival rate of 9%.^1^ Adequate surgical resection is patients’ only chance to be cured, often in combination with preoperative chemoradiotherapy or postoperative chemotherapy.^2,3,4^ At the time of diagnosis, only 25% to 30% of patients with PC are candidates for surgical management with curative intent.^3^

Although minimally invasive techniques have revolutionized many surgical procedures, it is far from generally accepted in PC surgery. Minimally invasive pancreatic surgery (MIPS; laparoscopic or robotic) has gained popularity in the last decade, albeit for tumor types other than PC. Except for a few expert centers, most reports come from low-volume centers performing less than 20 MIPS procedures for PC a year.^5,6,7^ Nonetheless, there is growing evidence, mostly from cohort studies, that short-term outcomes after MIPS may be comparable with those after open pancreatic surgery (OPS).^8,9,10^ To date, only few high-volume centers have reported on long-term oncologic outcomes after MIPS for PC, but none of them have shown superior long-term overall survival (OS) in favor of MIPS.^5,11^ The aim of this study was to compare overall survival (OS) and disease-free survival (DFS) rates after laparoscopic and open surgery for resectable or borderline resectable PC in a high-volume pancreatic cancer referral center.

## Methods

### Data Acquisition

A retrospective analysis of a prospectively maintained electronic database was performed for this comparative effectiveness study. This database contains patient data registered daily by means of the electronic patient record (EPR) system. Patients were selected based on clinical pathway labeling and surgical activity codes. Based on the unique patient identifier and procedure date or on the patient stay identifier, the study population data set was extended with clinical data. Mortality dates were retrieved either from the EPR system (in-hospital deaths) or from the central Federal Public Service Public Health data platform (deaths outside the hospital) based on patients’ unique national registry number. Mortality dates within this platform are updated daily and synchronized with the EPR system.

### Ethical Approval

The institutional medical ethics committee waived the need to review the study and the requirement for informed consent due to its retrospective nature. The study was performed in accordance with the principles of the Helsinki declaration^12^ and the General Data Protection Regulation.

### Patients

Between January 2010 and December 2019, 519 consecutive patients underwent pancreatic resection with curative intent for pathology-proven pancreatic ductal adenocarcinoma. Patients treated for oligometastatic disease (n = 21) were excluded from the study. After surgery, patients were followed up until death or until study closure in September 2021. Median (IQR) duration of follow-up was 56.8 (32.7-84.2) months. Two experienced pancreatic surgeons (B.T. for MIPS and OPS; R.A. for OPS) performed all surgical procedures. Both surgeons were considered to have passed their learning curve, which was defined as having performed more than 50 OPS for PC and more than 50 minimally invasive pancreaticoduodenectomies for any type of pancreatic head or periampullary tumor.^7^

### Procedures

Analysis was performed on an intention-to-treat basis. Patients were treated according to the latest National Comprehensive Cancer Network guidelines at the time of surgery. Patient and surgery characteristics are shown in Table 1. After propensity score matching, surgical procedures consisted of pancreaticoduodenectomy (157 OPS; 146 MIPS), distal pancreatectomy (37 OPS; 48 MIPS), or total pancreatectomy (4 OPS; 4 MIPS). Simultaneous vascular resection due to suspected tumor involvement was performed in 42 of 198 patients (21.2%) undergoing OPS vs 36 of 198 (18.2%) undergoing MIPS (*P* > .99). The conversion rate to open surgery was 10.6% (21 of 198) in MIPS. The most frequent reason for conversion was vascular involvement (13 patients). Other reasons were bleeding (n = 3), anatomical difficulties (n = 3), and excessive CO_2_ retention (n = 2) due to pneumoperitoneum.

**Table 1.** Patient and Surgery Characteristics

Characteristic	Without propensity score matching			With propensity score matching		
	Patients, No. (%)		*P* value	Patients, No. (%)		*P* value
	OPS (n = 300)	MIPS (n = 198)		OPS (n = 198)	MIPS (n = 198)	
Sex						
Male	140 (46.7)	89 (44.9)	.71	94 (47.5)	89 (44.9)	.69
Female	160 (53.3)	109 (55.1)		104 (52.5)	109 (55.1)	
Age, median (range), y	66 (37-85)	68 (32-87)	.34	67 (39-84)	68 (32-87)	.75
ASA score						
1	7 (2.3)	5 (2.5)	.28	6 (3.0)	5 (2.5)	.84
2	147 (49.0)	81 (40.9)		89 (44.9)	81 (40.9)	
3	143 (47.7)	111 (56.1)		102 (51.5)	111 (56.1)	
4	3 (1)	1 (0.5)		1 (0.5)	1 (0.5)	
BMI, median (range)	24.4 (17.0-50.5)	24.9 (16.5-38.9)	.73	24.4 (17.0-50.5)	24.9 (16.5-38.9)	.64
Diabetes						
No	230 (76.7)	150 (75.8)	.47	147 (75.2)	150 (75.8)	.59
Oral antidiabetic therapy	47 (15.7)	36 (18.2)		32 (16.2)	36 (18.2)	
Insulin therapy	23 (7.7)	12 (6.1)		17 (8.6)	12 (6.1)	
Exocrine pancreatic insufficiency	7 (2.3)	6 (3.0)	.77	5 (2.5)	6 (3.0)	>.99
Type of surgery						
Pancreaticoduodenectomy	229 (76.3)	146 (73.7)	.28	157 (79.3)	146 (73.7)	.44
Pancreatectomy						
Distal	59 (19.7)	48 (24.2)		37 (18.7)	48 (24.2)	
Total	12 (4.0)	4 (2.0)		4 (2.0)	4 (2.0)	
Simultaneous vascular resection	77 (25.7)	36 (18.2)	.06	42 (21.2)	36 (18.2)	.53
Type of vascular resection						
SMV/PV	67 (22.3)	32 (16.2)	.27	39 (17.2)	32 (16.2)	.84
Celiac trunk artery	5 (1.7)	2 (1.0)		2 (1.0)	2 (1.0)	
SMV/PV and celiac trunk artery	5 (1.7)	2 (1.0)		1 (0.5)	2 (1.0)	
Type of vascular reconstruction						
Primary suture of wedge-resection	23 (7.7)	17 (8.6)	.003	12 (6.1)	17 (8.6)	.03
Primary end-to-end reconstruction of segmental resection	17 (5.7)	4 (2.0)		9 (4.5)	4 (2.0)	
Graft interposition	32 (10.7)	7 (3.5)		19 (9.6)	7 (3.5)	
Duration of surgery, median (range), min	220 (74-450)	270 (70-570)	<.001	211 (74-450)	270 (70-570)	<.001
Blood loss intra-operative, median (range), mL	300 (0-2500)	10 (0-1300)	<.001	300 (0-1500)	10 (0-1300)	<.001
Blood transfusion						
Intraoperative	108 (36.0)	15 (7.6)	<.001	64 (32.3)	15 (7.6)	<.001
Postoperative	100 (34.3)	22 (15.8)	<.001	60 (31.1)	22 (15.8)	.002

Abbreviations: ASA, American Society of Anesthesiologists; BMI, body mass index (calculated as weight in kilograms divided by height in meters squared); MIPS, minimally invasive pancreatic surgery; OPS, open pancreatic surgery; PV portal vein; SMV, superior mesenteric vein.

## Outcomes

Survival (OS and DFS) rates, cancer recurrence (locoregional and metastases) rates, surgical resection margins, and the number of lymph nodes retrieved were studied as oncologic outcomes. The magnitude of tumor-free resection margins was defined as R0 (>1 mm), R1 indirect (≤1 mm), or R1 direct (no tumor-free margin).^13^ Tumor staging was determined using the Union for International Cancer Control TNM classification system, eighth edition.

The number and type of postoperative complications were allocated to surgical site and nonsurgical site complications. Rates and types of postoperative pancreatic fistula, pancreatic hemorrhage, biliary fistula, and delayed gastric emptying were defined and recorded according to the International Study Group of Pancreatic Surgery guidelines.^14,15,16,17^ Severity of postoperative complications was defined according to the Clavien-Dindo therapy-oriented severity grading system of complications.^18^

### Statistical Analysis

Survival outcomes were analyzed using the Cox proportional hazards model, and results are presented as hazard ratios (HRs). Surgical resection margin was analyzed as an ordinal variable (R1 direct worse than R1 indirect worse than R0), using a proportional odds model, after testing the underlying proportional odds assumption. These results are presented as odds ratios (ORs). The number of lymph nodes retrieved was analyzed as a continuous variable using a linear model, with results presented as mean differences and the symmetry of the distribution was inspected visually.

Corrections for baseline group differences were performed by means of propensity score matching.^19,20^ Each patient in the MIPS group was individually matched to a patient in the OPS group based on similarity of the propensity score. Variables used for the propensity score model were TNM stage, tumor dimension (pT), lymph node status (pN), type of operation, simultaneous vascular resection, neoadjuvant chemotherapy, adjuvant chemotherapy within 3 months after surgery, sex, age, and American Society of Anesthesiologists score. For survival outcomes, additional corrections were made for year of surgery and type of adjuvant chemotherapy.

Data analyses of the matched sample were performed considering clustering due to matching case with control participants. Conditional logistic regression analysis was performed for binary outcomes, linear mixed models were used for continuous outcomes with a random cluster effect, and time-to-event data were analyzed using Cox models with estimation using the robust sandwich estimator of Lin and Wei.^21^ Follow-up summary statistics were based on the Kaplan-Meier estimate of potential follow-up,^22^ ie, a median (IQR) of 84.2 (58.2-102.6) months in OPS and 39.1 (28.5-63.5) months in MIPS (*P* < .001). Data analysis was conducted from March to October 2022.

Categorical variables were reported as frequencies and percentages. Group differences were analyzed using the Fisher exact test. Continuous variables were described as median and range, and group differences analyzed using the Mann-Whitney *U* test. Survival rates were estimated using the Kaplan-Meier method. All reported *P* values were 2-sided. All tests were performed at a 2-sided 5% significance level. Analyses were performed using SAS software version 9.4 (SAS Institute).

## Results

After propensity score matching, 198 patients who underwent MIPS (89 [44.9%] men; median [range] age, 68 [32-87] years) were compared with 198 control participants who underwent OPS (94 [47.5%] men; median [range] age, 67 [39-84] years) (Table 1). Neoadjuvant chemotherapy was administered for borderline resectable PC in 23 patients (11.6%) before MIPS and in 28 (14.1%) before OPS (*P* = .55). The type of neoadjuvant chemotherapy was similar in MIPS and OPS (FOLFIRINOX: 16 [69.6%] vs 15 [53.6%]; gemcitabine: 7 [30.4%] vs 13 [46.4%]; *P* = .27).

### Survival

In the propensity score–matched sample, median OS in MIPS was 30.7 (95% CI, 26.2-36.8) months compared with 20.3 (95% CI, 17.6-23.5) months in OPS (HR, 0.70; 95% CI, 0.56-0.87; *P* = .002). Overall survival rates at 1, 3, and 5 years after MIPS vs OPS were 80.3% (95% CI, 74.0%-85.2%) vs 72.6% (95% CI, 65.8%-78.3%), 44.0% (95% CI, 36.3%-51.5%) vs 27.5% (95% CI, 21.4%-33.9%), and 23.3% (95% CI, 15.5%-32.1%) vs 17.4% (95% CI, 12.2%-23.4%), respectively (Figure, A). After additional correction for year of surgery (HR, 0.74; 95% CI, 0.57-0.96; *P* = .02) and type of adjuvant chemotherapy (FOLFIRINOX vs other) (HR, 0.71; 95% CI, 0.56-0.90; *P* = .005), these differences remained statistically significant in favor of MIPS (Table 2). Median DFS after MIPS vs OPS was 14.8 (95% CI, 11.8-17.0) months vs 10.7 (95% CI, 9.01-12.11) months (HR, 0.71; 95% CI, 0.57-0.89; *P* = .003). DFS rates at 1, 3, and 5 years after MIPS vs OPS were 56.6% (95% CI, 49.4%-63.1%) vs 43.4% (95% CI, 36.5%-50.2%), 22.6% (95% CI, 16.5%-29.3%) vs 14.0% (95% CI, 9.6%-19.2%), and 18.5% (95% CI, 12.3%-25.7%) vs 10.8% (95% CI, 6.9%-15.8%), respectively (Figure, B). After additional correction for year of surgery (HR, 0.77; 95% CI, 0.59-0.99; *P* = .04) and type of adjuvant chemotherapy (FOLFIRINOX vs other) (HR, 0.72; 95% CI, 0.57-0.92; *P* = .009), these differences remained statistically significant in favor of MIPS (Table 2).

**Figure.**
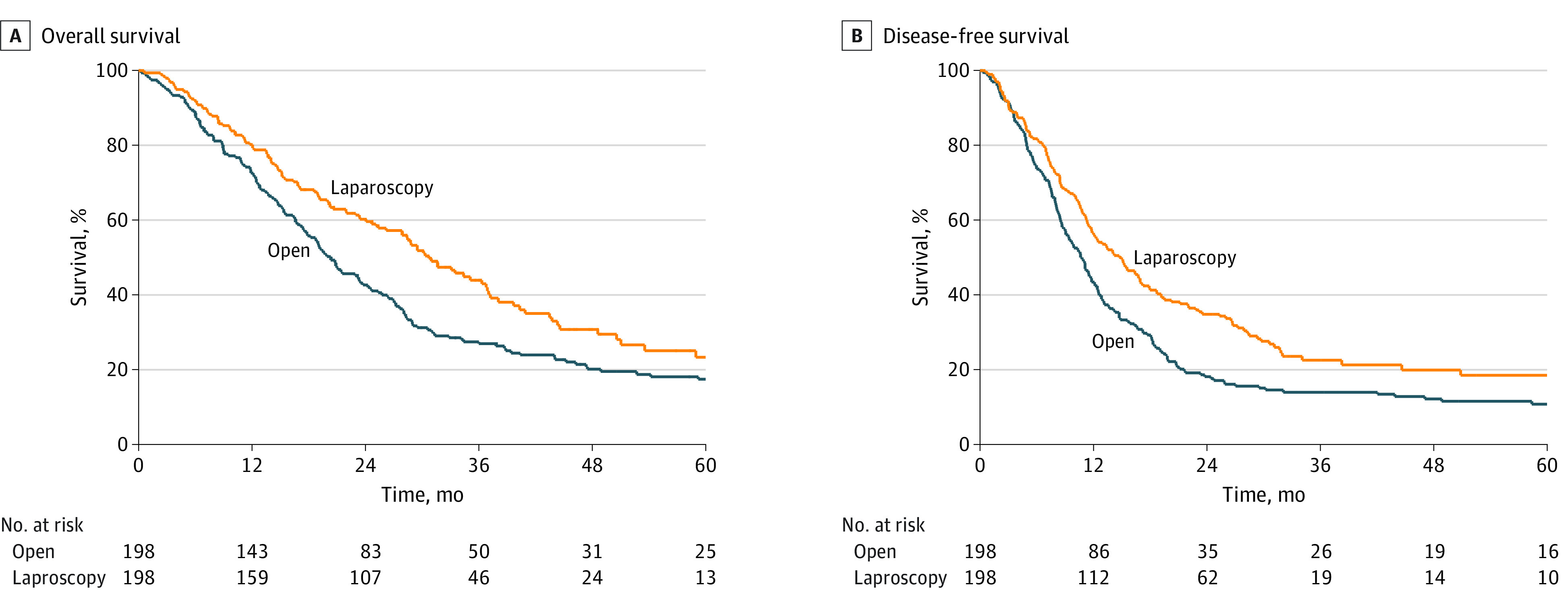
Overall and Disease-Free Survival After Laparoscopic and Open Pancreatic Surgery for Pancreatic Adenocarcinoma A, Laparoscopy vs open surgery: hazard ratio, 0.70; 95% CI, 0.56 to 0.87; *P* = .002. B, Laparoscopy vs open surgery: hazard ratio, 0.71; 95% CI, 0.57-0.89; *P* = .003.

**Table 2.** Results for Overall and Disease-Free Survival After Minimally Invasive vs Open Surgery for Pancreatic Adenocarcinoma

Correction	Overall survival				Disease-free survival			
	HR (95% CI)	*P* value	No.		HR (95% CI)	*P* value	No.	
			Patients	Events			Patients	Events
None	0.69 (0.56-0.86)	.001	498	380	0.74 (0.60-0.90)	.003	498	421
Propensity score matching	0.70 (0.56-0.87)	.002	396	290	0.71 (0.57-0.89)	.003	396	330
Propensity score matching and year of surgery	0.74 (0.57-0.96)	.02	396	290	0.77 (0.59-0.99)	.04	396	330
Propensity score matching and type of adjuvant chemotherapy	0.71 (0.56-0.90)	.005	396	290	0.72 (0.57-0.92)	.009	396	330

Abbreviation: HR, hazard ratio.

In the propensity score–matched sample, metastases occurred in 118 patients (59.6%) after MIPS and in 142 (71.7%) after OPS (*P* = .005). Median metastasis-free survival after MIPS was 18.9 (95% CI, 14.9-26.3) months vs 13.4 (95% CI, 12.0-17.7) months after OPS (*P* = .003). Locoregional cancer recurrence occurred in 41 patients (20.7%) after MIPS and in 48 (24.2%) after OPS (*P* = .32). Median time to locoregional cancer recurrence after MIPS or OPS was not reached at the time of study closure (*P* = .17). In the propensity score–matched sample, postoperative 30-day and 90-day mortality rates were 0.5% and 3.0% after MIPS vs 1.5% and 5.0% after OPS (*P* = .62 and *P* = .44, respectively).

### Oncologic Outcomes

In the propensity score–matched sample, tumor characteristics were similar in MIPS and OPS groups and are shown in Table 3. Tumor-free resection margin was larger than 1 mm in 98 patients (49.5%) in the MIPS group vs 115 (58.1%) in the OPS group. The rate of microscopic tumor involvement of surgical resection margins (R1 direct or R1 indirect) was 100 (50.5%) in the MIPS group and 83 (41.9%) in the OPS group. Based on a proportional odds model accounting for propensity score matching, similar resection margin status was found in MIPS and OPS (OR, 1.26; 95% CI, 0.85-1.89; *P* = .25). The median (range) number of lymph nodes retrieved was 21 (1-51) in the MIPS group vs 20 (3-52) in the OPS group. Based on a linear model accounting for propensity score matching, the number of lymph nodes retrieved was similar in MIPS vs OPS (mean difference, 0.77; 95% CI, −1.19 to 2.74; *P* = .44).

**Table 3.** Tumor Characteristics

Characteristic	Without propensity score matching			With propensity score matching		
	Patients, No. (%)		*P* value	Patients, No. (%)		*P* value
	OPS (n = 300)	MIPS (n = 198)		OPS (n = 198)	MIPS (n = 198)	
TNM stage						
1a	34 (11.3)	18 (9.1)	.52	18 (9.1)	18 (9.1)	.84
1b	40 (13.3)	37 (18.7)		30 (15.1)	37 (18.7)	
2a	14 (4.7)	7 (3.5)		10 (5.0)	7 (3.5)	
2b	106 (35.3)	69 (34.8)		74 (37.4)	69 (34.8)	
3	106 (35.3)	67 (33.8)		66 (33.3)	67 (33.8)	
Tumor diameter, median (range), mm	30 (5-170)	30 (4-90)	.61	30 (8-170)	30 (4-90)	.47
T status						
1	65 (21.7)	31 (15.7)	.07	35 (17.7)	31 (15.7)	.95
2	164 (54.7)	127 (64.1)		122 (61.6)	127 (64.1)	
3	58 (19.3)	37 (18.7)		38 (19.2)	37 (18.7)	
4	13 (4.3)	3 (1.5)		3 (1.5)	3 (1.5)	
N status						
0	91 (30.3)	63 (31.8)	.81	59 (29.8)	63 (31.8)	.84
1	113 (36.7)	69 (34.9)		75 (37.9)	69 (34.9)	
2	96 (32.0)	66 (33.3)		64 (32.3)	66 (33.3)	
Resection margin status						
R0	163 (54.3)	98 (49.5)	.10	115 (58.1)	98 (49.5)	.07
R1 indirect	81 (27.0)	71 (35.9)		50 (25.2)	71 (35.9)	
R1 direct	56 (18.7)	29 (14.6)		33 (16.7)	29 (14.6)	
Lymph nodes retrieved, median (range), No.	19 (3-61)	21 (1-51)	.08	20 (3-52)	21 (1-51)	.21
Neoadjuvant chemotherapy	47 (15.7)	23 (11.6)	.24	28 (14.1)	23 (11.6)	.55
Type neoadjuvant chemotherapy						
Gemcitabine	18 (6.0)	7 (3.5)	.60	13 (46.4)	7 (30.4)	.27
FOLFIRINOX	29 (9.7)	16 (8.1)		15 (53.6)	16 (69.6)	
Adjuvant chemotherapy	205 (68.3)	155 (78.3)	.02	149 (75.2)	155 (78.3)	.55
Type adjuvant chemotherapy						
Gemcitabine	185 (61.7)	108 (54.6)	<.001	138 (92.6)	108 (69.7)	<.001
FOLFIRINOX	15 (5.0)	40 (20.2)		7 (4.7)	40 (25.8)	
Oxaliplatin	5 (1.6)	7 (3.5)		4 (2.7)	7 (4.5)	

Abbreviations: MIPS, minimally invasive pancreatic surgery; OPS open pancreatic surgery; R0, tumor-free resection margin larger than 1 mm; R1 direct, no tumor-free resection margin; R1 indirect, tumor-free resection margin of 1 mm or less.

More patients received adjuvant chemotherapy within 3 months after MIPS vs OPS (155 [78.3%] vs 149 [68.8%]; *P* = .55). The type of adjuvant chemotherapy was based on the standard of care at the time of surgery and was different in MIPS vs OPS (FOLFIRINOX: 40 [25.8%] vs 7 [4.7%]; *P* < .001; gemcitabine: 108 [69.7%] vs 138 [64.6%]; *P* = .04; oxaliplatin: 7 [4.5%] vs 4 [2.7%]; *P* = .09).

### Clinical Outcomes

In the propensity score–matched sample, median (IQR) duration of surgery was 270 (215-330) minutes in the MIPS group and 210 (180-245) minutes in the OPS group (*P* < .001). Blood transfusion was given intraoperatively in 15 patients (7.6%) during MIPS vs 64 (32.3%) during OPS (*P* < .001) and postoperatively in 22 (15.8%) after MIPS vs 60 (31.1%) after OPS (*P* = .002). Clinical outcomes are shown in Table 4. Postoperative complications were observed in 105 patients (53.0%) who underwent MIPS vs 120 (60.6%) who underwent OPS (*P* = .15). The rate of surgical site complications (88 [44.4%] vs 87 [43.9%]; *P* > .99) was similar, whereas the rate of non–surgical site complications (35 [17.7%] vs 56 [28.3%]; *P* = .02) differed in favor of MIPS. Similar rates of severe complications (therapy-oriented severity grading system grade ≥3a) and reoperations were observed in MIPS and OPS groups (25 [12.6%] vs 28 [14.1%]; *P* = .77 and 17 [8.6%] vs 16 [8.1%]; *P* > .99, respectively).

**Table 4.** Clinical Outcomes After MIPS and OPS for Patients With Pancreatic Adenocarcinoma

Outcome	Without propensity score matching			With propensity score matching		
	Patients, No. (%)		*P* value	Patients, No. (%)		*P* value
	OPS (n = 300)	MIPS (n = 198)		OPS (n = 198)	MIPS (n = 198)	
Postoperative mortality						
30-d	6 (2.0)	1 (0.5)	.25	3 (1.5)	1 (0.5)	.62
90-d	17 (5.7)	6 (3.0)	.27	10 (5.0)	6 (3.0)	.44
Complications	180 (60.0)	105 (53.0)	.06	120 (60.6)	105 (53.0)	.15
Location of complications, site						
Surgical	134 (44.7)	88 (44.4)	.79	87 (43.9)	88 (44.4)	>.99
Nonsurgical	85 (28.3)	35 (17.7)	.01	56 (28.3)	35 (17.7)	.02
Severe complications, ie, Clavien-Dindo classification grade 3 or greater	50 (16.7)	25 (12.6)	.22	28 (14.1)	25 (12.6)	.77
Clinical pancreatic fistula	44 (14.7)	44 (22.2)	.03	25 (12.6)	44 (22.2)	.02
Hemorrhage	26 (8.7)	15 (7.6)	.74	16 (8.1)	15 (7.6)	>.99
Biliairy fistula	8 (2.7)	5 (2.5)	>.99	6 (3.0)	5 (2.5)	>.99
Delayed gastric emptying	68 (22.7)	11 (5.6)	<.001	49 (24.7)	11 (5.6)	<.001
Reoperation	28 (9.3)	17 (8.6)	.75	16 (8.1)	17 (8.6)	>.99
Readmission	11 (3.7)	30 (15.2)	<.001	9 (4.5)	30 (15.2)	<.001
Length of hospital stay after surgery, median (range), d	17 (4-185)	11 (5-70)	<.001	17 (7-85)	11 (5-70)	<.001

Abbreviations: MIPS, minimally invasive pancreatic surgery; OPS, open pancreatic surgery.

Clinical postoperative pancreatic fistula (grade B or C) ware observed in 44 patients (22.2%) after MIPS and in 25 (12.6%) after OPS (*P* = .02). The rates of hemorrhage (15 [7.6%] vs 16 [8.1%]; *P* > .99) and biliary fistula (5 [2.5%] vs 6 [3.0%]; *P* > .99) were similar in both groups. Fewer patients developed delayed gastric emptying after MIPS compared with OPS (11 [5.6%] vs 49 [24.7%]; *P* < .001). Median (IQR) length of hospital stay was 11 (8-16) days after MIPS vs 17 (13-23) days after OPS (*P* < .001). The readmission rate within 30 days after discharge from hospital was 15.2% (30 patients) after MIPS vs 4.5% (9 patients) after OPS (*P* < .001).

## Discussion

### Survival

In this study, long-term survival of patients treated for pancreatic cancer was significantly better after minimally invasive than after standard open surgery. Fewer patients developed metastases after minimally invasive vs open surgery, and it took longer to develop them. Median OS was 30.7 months, and the 5-year OS rate was 23.3% after MIPS vs 20.3 months and 17.4% after OPS. Also, median and 5-year DFS were in favor of MIPS vs OPS (14.8 months and 18.5% vs 10.7 months and 10.8%). To our knowledge, this is the first study reporting on superior long-term OS and DFS rates after MIPS vs OPS for patients with PC.

Systemic chemotherapy in the adjuvant and/or neoadjuvant setting substantially improves long-term survival of patients with (borderline) resectable PC.^3,23^ Approximately 13% of patients in this study received neoadjuvant systemic chemotherapy for borderline resectable PC, equally distributed between MIPS and OPS. The type of neoadjuvant chemotherapy (FOLFIRINOX or gemcitabine) was also similar in both groups. After propensity score matching, similar numbers of patients (approximately 75%) received adjuvant chemotherapy, but the type of adjuvant chemotherapy differed significantly between MIPS and OPS. More patients received the more effective type of chemotherapy (FOLFIRINOX) after MIPS (26%) than after OPS (5%). Most patients received gemcitabine as adjuvant chemotherapy, both after MIPS (70%) and OPS (93%). These findings are explained by the change of the adjuvant chemotherapy regimen from gemcitabine to FOLFIRINOX in 2018 to 2019, the last year when patients were included in this study.^3^ As the type of adjuvant chemotherapy and the growing expertise in MIPS during the course of the study may explain survival differences in both groups, additional statistical corrections were applied for year of surgery and type of adjuvant chemotherapy. After these corrections, MIPS was associated with better OS and DFS compared with OPS.

Surgical volume and expertise have an impact on both clinical and oncologic outcomes in patients treated for borderline resectable and resectable PC.^24^ Few centers with an annual volume of more than 20 MIPS procedures for PC have published their outcomes, and available data are too heterogenous to allow comparative analysis.^25^ After pancreatic surgery, the absolute differences in adjusted mortality between the lowest and highest volume hospitals were found to be among the highest.^26^ The annual average surgical volume in the current study was more than 50 pancreatic resections for PC, which reflects available expertise. In the present study, the surgeons were already experienced before starting the study and showed a sustained high level of surgical adequacy within the study. Postoperative mortality rates were low and similar in both MIPS and OPS groups and thus cannot explain long-term survival differences. In pancreatic cancer, the magnitude of tumor-free resection margins, with a cutoff at 1 mm from the surgical resection margins, is associated with long-term survival.^13^ In the present study, R0-resection margin rates were similar in the MIPS and OPS groups and similar to those in literature.^5,6,8,9,13^ Simultaneous vascular resection to obtain R0-resection margins for suspected major vascular involvement was performed in 19.7% of all patients, similar in MIPS (18.2%) and OPS (21.2%). Also, the type of vascular resection was similar in MIPS and OPS, which illustrates both groups include patients with PC with similar locoregional tumor extension and vascular involvement. Besides the surgical resection margin status, the number of lymph nodes retrieved is a surrogate for the adequacy of surgical oncology. In the current study, the lymph node yield was similarly high in MIPS (median, 21) and OPS (median, 20). Tumor- and patient-related factors also have an impact on survival. In this study TNM stage, pT, pN, sex, age, and American Society of Anesthesiologists score were used for propensity score matching and similarly represented in both treatment groups.

### Surgical Trauma Promoting Cancer Progression

Because surgical trauma of the standard open approach is more extensive than that of minimally invasive surgery, locoregional and systemic effects are more favorable after minimally invasive compared with open surgery.^27^ In lung and colon cancer, but not in other cancers, better survival has been reported after minimally invasive compared with open surgery.^28,29,30,31,32^ Much research has concentrated on perioperative locoregional and systemic biologic perturbations involved in cancer recurrence and postoperative development of metastasis.^33,34,35^ Even in early stages of cancer, circulating tumor cells are detectable both before and after surgical resection of PC, capable of causing cancer recurrence.^36^ Surgical trauma induces the release of a number of neuro-endocrine, angiogenic, and growth factors. Release of catecholamines and prostaglandins by damaged tissue can activate tumor cells as they bear surface receptors for these molecules.^36,37^ They and other modulating factors cause immunosuppression and favor metastasis-promoting processes and cancer progression.^33,34,35^ Platelets, activated during surgery to achieve hemostasis, can form a shell around circulating tumor cells and protect them from destruction by the host immune system.^33^ Additional immunosuppression is caused by intraoperative hypothermia that might occur more frequently in open procedures.^38^ Hypothermia can cause impaired blood clotting and—secondary to more bleeding—increase the need for blood transfusion.^39^ Finally, the association between perioperative blood transfusion and its deleterious effect on the oncologic outcome was shown in a Cochrane review of 36 studies.^40^ In the current study, patients in OPS had significantly more transfusion need both intraoperatively and postoperatively.

### Limitations

The limitation of this study is its retrospective and thus nonrandomized nature. For now, we believe it is hard to conduct a randomized trial in which participating centers have passed their learning curve on minimally invasive PC surgery and perform at least 50 procedures a year. Ideally, this trial should also be stratified for variables such as TNM stage, simultaneous vascular resection, type of surgical procedure, and neoadjuvant and adjuvant chemotherapy. We took these variables into account and used them in the propensity score–based comparative analysis and additionally corrected for year of surgery and the type of adjuvant chemotherapy, aiming to minimize the effect of bias. However, it is possible that some not-yet measurable aspect of patient selection or surgeon judgment was at play and was not considered in the propensity score–matched analysis.

## Conclusions

In this study of 396 patients with borderline resectable and resectable pancreatic adenocarcinoma, MIPS was associated with better OS and DFS than OPS. Centralization of MIPS for PC should be stimulated, and pancreatic surgeons should be encouraged to pass the learning curve before implementing MIPS for PC in daily clinical practice.

## Notes

Supplement.
Data Sharing Statement
