# Supplementary material for: Survival After Minimally Invasive vs Open Surgery for Pancreatic Adenocarcinoma
Source: JAMA Netw Open. 2022 Dec 22;5(12):e2248147. doi: 10.1001/jamanetworkopen.2022.48147 (PMC9857028; doi:10.1001/jamanetworkopen.2022.48147)
Supplement: Supplement. — Data Sharing Statement [file jamanetwopen-e2248147-s001.pdf]

Data Sharing Statement

Topal. Survival After Minimally Invasive vs Open Surgery for Pancreatic Adenocarcinoma. *JAMA Netw Open*. Published December 22, 2022. doi:10.1001/jamanetworkopen.2022.48147

Data
Data available: No

Additional Information
Explanation for why data not available: GDPR
